# Age-associated miRNA Alterations in Skeletal Muscle from Rhesus Monkeys reversed by caloric restriction

**DOI:** 10.18632/aging.100598

**Published:** 2013-09-09

**Authors:** Evi M. Mercken, Elisa Majounie, Jinhui Ding, Rong Guo, Jiyoung Kim, Michel Bernier, Julie Mattison, Mark R. Cookson, Myriam Gorospe, Rafael de Cabo, Kotb Abdelmohsen

**Affiliations:** ^1^ Experimental Gerontology Section, Translational Gerontology Branch, National Institute on Aging, National Institutes of Health, Baltimore, MD 21224, USA; ^2^ Cell Biology and Gene Expression Section, Laboratory of Neurogenetics, National Institute on Aging, National Institutes of Health, Bethesda, MD 20892, USA; ^3^ Laboratory of Genetics, National Institute on Aging, National Institutes of Health, Baltimore, MD 21224, USA

**Keywords:** gene expression, posttranscriptional gene regulation, muscle aging, muscle diseases

## Abstract

The levels of microRNAs (miRNAs) are altered under different conditions such as cancer, senescence, and aging. Here, we have identified differentially expressed miRNAs in skeletal muscle from young and old rhesus monkeys using RNA sequencing. In old muscle, several miRNAs were upregulated, including miR-451, miR-144, miR-18a and miR-15a, while a few miRNAs were downregulated, including miR-181a and miR-181b. A number of novel miRNAs were also identified, particularly in old muscle. We also examined the impact of caloric restriction (CR) on miRNA abundance by reverse transcription (RT) followed by real-time, quantitative (q)PCR analysis and found that CR rescued the levels of miR-181b and chr1:205580546, and also dampened the age-induced increase in miR-451 and miR-144 levels. Our results reveal that there are changes in expression of known and novel miRNAs with skeletal muscle aging and that CR may reverse some of these changes to a younger phenotype.

## INTRODUCTION

Aging is a major risk factor for sarcopenia, the degenerative loss of skeletal muscle mass and strength. Skeletal muscle is central to the quality of human life through its involvement in several functions such as posture maintenance, movement, breathing, and metabolism [1, 2]. Age-related alterations in the composition of skeletal muscle are linked to functional limitations, disability and metabolic disorders. Alterations in muscle damage and repair during aging can have deleterious consequences that lead to muscle degeneration and inflammation [3]; most of the age-related declines in muscle homeostasis and function can be prevented by caloric restriction (CR) in laboratory animals [4]. Changes in gene expression critically govern the age-related alterations in muscle mass and function. The underlying mechanisms that elicit muscle aging include oxidative stress, mitochondrial dysfunction (structural integrity and biogenesis), apoptosis, autophagy, and reduction in protein synthesis [1, 5].

MicroRNAs (miRNAs) regulate gene expression by recruiting the RNA-induced silencing complex (RISC) to a target mRNA with which it shares partial complementarity, causing a reduction in the stability of the mRNA and/or its rate of translation [6]. The expression of miRNAs is altered in many biological processes such as development, cell division, differentiation, and senescence, as well as in pathological conditions like cancer, cardiovascular disease, amytrophic lateral sclerosis (ALS), and *muscular dystrophy* [7-17]. The relevance of miRNAs in disease development, muscle aging, and progression and prognosis of skeletal muscle diseases is not fully understood. The profiling of miRNAs in aged tissues can provide direct links between aging, age-dependent regulation of miRNA abundance, and the involvement of miRNAs in normal aging and age-related diseases. In this study, changes in miRNAs in skeletal muscle from rhesus monkeys of different ages were assessed using RNA sequencing (RNA-Seq). Our results showed clear differences in muscle miRNA levels when comparing old and young animals, and that CR influences these age-induced changes in miRNA expression. Novel miRNAs were also identified in muscle of old and young rhesus monkeys, which could potentially be expressed in human skeletal muscle. Together, our study provides further support for the role of miRNAs in skeletal muscle aging and reveals the impact of CR on miRNA expression.

## RESULTS

### Global miRNA changes between young and old skeletal muscles from rhesus monkeys

**Figure 1:**
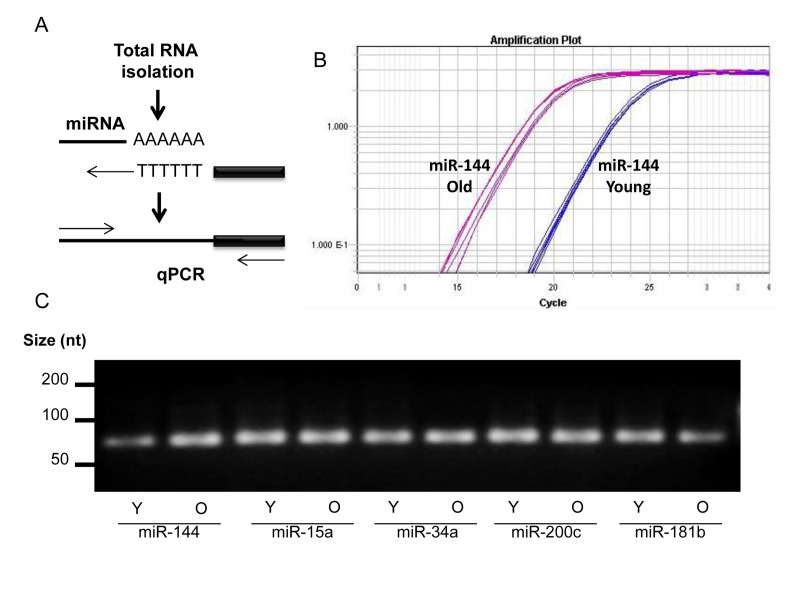
miRNA detection **(A)** Total RNA was extended by the addition of a poly(A) tail to the 3' end using poly(A) polymerase. Hybridization of the adaptor was followed by reverse transcription to synthesize first-strand. RT-qPCR was then performed using a universal reverse primer and miRNA-specific forward primer. **(B)** An example of amplification curve of miR-144 in young and old tissues. **(C)** After amplification, the end-product was size-fractionated and visualized by ethidium bromide agarose gels.

Sequencing of miRNAs was performed using RNA isolated from skeletal muscle tissues of four young male (6 years) and four old male (26.8 years) rhesus monkeys. miRNA reads were analyzed using miRDeep2 software and mapped to the rhesus genome. This analysis revealed differentially expressed miRNAs (Table S1). For instance, miR-451, miR-144, miR-15a, miR-15b, miR18a, and miR-34a were significantly more abundant in skeletal muscle tissues from old compared to young rhesus (Table 1, *top*), p values of ≤0.005, while other miRNAs, such as miR 181a, miR-181b, miR-1323, and miR-489, were significantly lower in skeletal muscle tissues from oldrhesus (Table 1, *bottom*), p values of ≤0.005. The changes in a subset of miRNAs were validated by real-time reverse transcription PCR (RT-qPCR) (Fig. 1A). An example of successful amplification of miR-144 from young and old skeletal muscle tissues is shown in Fig. 1B. In addition, end-products of the RT-qPCR reactions after 40 cycles (and therefore not quantitative) were analyzed by electrophoresis through agarose gels to confirm that a single DNA species was amplified (Fig. 1C).

**Table 1:** Differentially expressed miRNAs in aged skeletal muscle tissues from rhesus monkeys RNA extracted from young and old monkeys was used for miRNA sequencing. p values of ≤0.005 are indicated.

miRNA ID	Fold Change	P-Value
mml-miR-451	84.750	0.001
mml-miR-144	53.594	0.000
mml-miR-129	46.824	0.045
mml-miR-200c	30.109	0.022
mml-miR-942	18.402	0.025
mml-miR-141	18.233	0.002
mml-miR-142-3p	11.852	0.006
mml-miR-18a	10.082	0.000
mml-miR-106b	6.358	0.005
mml-miR-15b	4.606	0.046
mml-miR-215	4.307	0.011
mml-miR-223	4.290	0.000
mml-miR-194	4.194	0.001
mml-miR-409-5p	4.159	0.030
mml-miR-93	3.915	0.017
mml-miR-17-5p	3.501	0.004
mml-miR-495	3.448	0.042
mml-miR-32	3.402	0.020
mml-miR-19a	3.401	0.048
mml-miR-20a	3.276	0.003
mml-miR-1271	3.164	0.001
mml-miR-17	2.988	0.036
mml-miR-221	2.808	0.005
mml-miR-339	2.741	0.005
mml-miR-16	2.649	0.051
mml-miR-34a	2.623	0.020
mml-miR-15a	2.457	0.007
mml-miR-192	2.299	0.015
mml-miR-19b	1.963	0.044
mml-miR-29b	1.894	0.058
mml-miR-181b	0.521	0.056
mml-miR-181a	0.495	0.037
mml-miR-653	0.435	0.033
mml-miR-489	0.341	0.003
mml-miR-1323	0.283	0.001

### CR ameliorates age-mediated changes in miRNA levels in old skeletal muscle tissues

**Figure 2:**
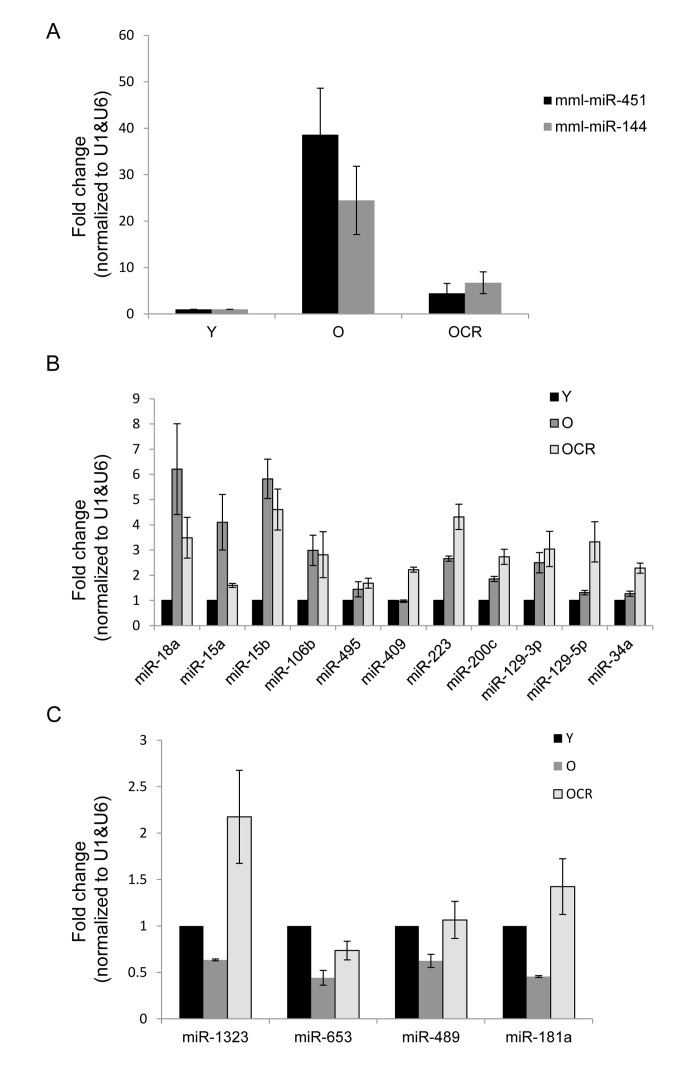
Validation of age-associated miRNA alterations and impact of CR **(A-C)** RT-qPCR analysis showing the abundance of the indicated miRNAs in young (Y), old (O), and old CR (OCR). RNAs from 9 Y samples, 7 O samples, and 9 OCR samples were used for this analysis and data was normalized to the average of U1 and U6 snRNA levels.

RT-qPCR analysis indicated that miR-451 and miR-144 levels increased by 35-fold and 25-fold, respectively, while other miRNAs such as miR-18a, miR-15a, and miR-15b showed modest changes in muscle tissues from old compared with young monkeys (Fig. 2A,B). In contrast, miR-181b, miR-1323, miR653, and miR-489 were less abundant in old versus young muscle tissues (Fig. 2C). The validation experiments included RNA samples from 9 monkeys (27.8 years, males) subjected to caloric restriction (CR) as previously described [53]. When the impact of CR on age-associated changes in miRNA levels was investigated, we found that CR significantly lowered age-mediated induction of miR-451 and miR-144 as well as the enhanced expression of miR-18a and miR-15a (Fig. 2A).

In contrast, the levels of miR-15b, miR-106b, miR-200c, and miR-129-5p in old tissues were not influenced by CR, while the expression of miR-223 appeared to be further enhanced (Fig. 2B). CR increased the abundance of miR-1323, which was downregulated in old muscle tissues; similarly, the age-associated reduction of miR-181b and miR-489 was reversed by CR, while miR-653 was only slightly influenced (Fig. 2C). Together, these data suggest that miRNA expression pattern changes in aged skeletal muscle tissues and CR may selectively influence the levels of a subset of these miRNAs.

### Age-altered miRNAs are involved in skeletal muscle development and disorders

**Figure 3:**
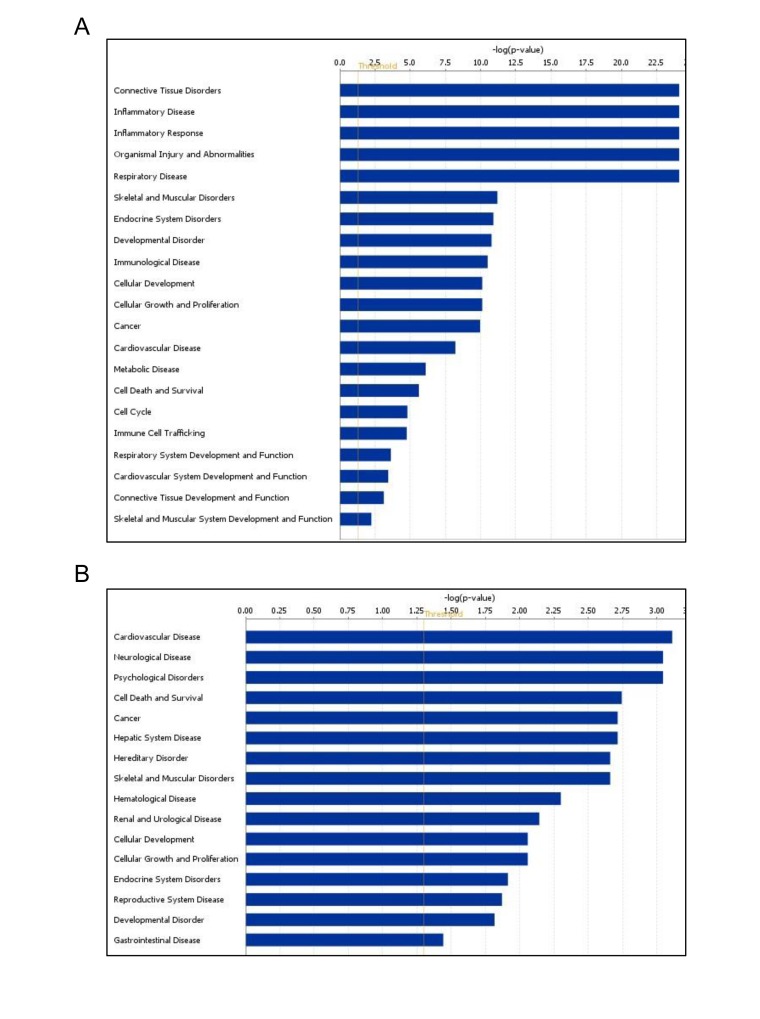
Age-associated miRNA alterations are involved in skeletal muscle development and disorders **(A,B)** Differentially expressed miRNAs shown in Table 1 were analyzed using Ingenuity Pathway Analysis (IPA). The analysis revealed that these miRNAs are linked to muscle development and disorders.

Using Ingenuity Pathways Analysis (IPA), we performed a comparative analysis of altered miRNAs between young and old skeletal muscle tissues to obtain a global view of the involvement of these miRNAs in biological processes. Interestingly, miRNAs upregulated in old tissues were found to be involved in connective tissue and skeletal muscle disorders, cell cycle, inflammatory diseases, inflammatory response, and skeletal and muscular system development and function (Fig. 3A). Moreover, downregulated miRNAs were involved in cardiovascular disease, cell death, and neurological and skeletal muscle disorders (Fig. 3B). These data indicate that the altered miRNAs in aged muscle are involved in several cellular processes and diseases while both subsets of miRNAs are associated with skeletal muscle disorders.

### Novel miRNAs revealed by RNA sequencing

**Figure 4:**
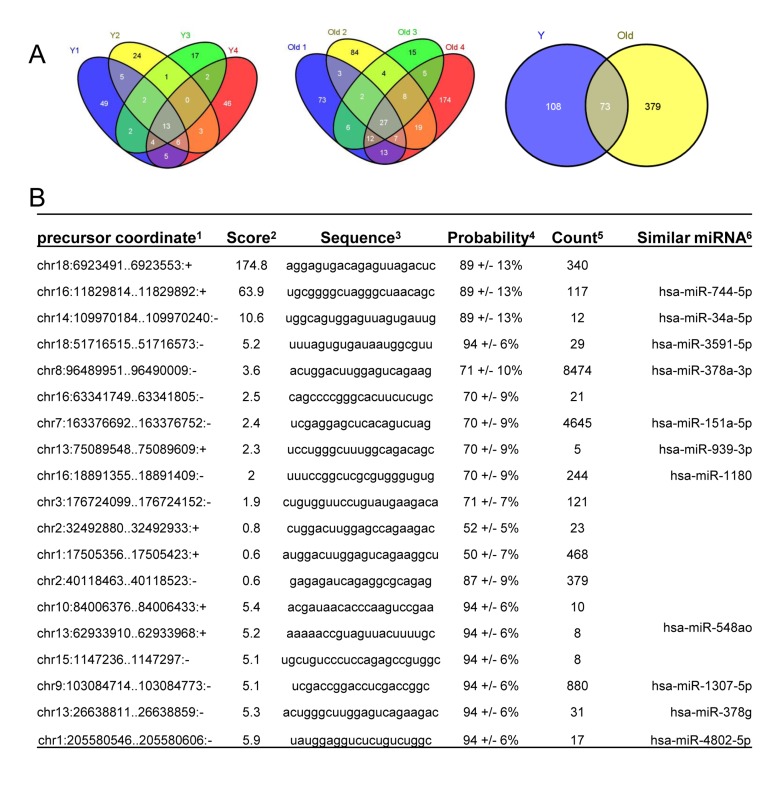
Identification of novel miRNAs in young and old skeletal muscle tissues from rhesus monkeys **(A)** Venn diagrams showing the number of miRNAs identified in young (Y) tissues (left), old (O) tissues (middle) and shared between young (Y) and old (O) tissues (right). **(B)**Partial list of novel miRNAs, ^1^location of the miRNA precursor in the human genome, ^2^miRDeep2 score represents the log-odds probability of a sequence being genuine miRNA precursor versus the probability that it is a background hairpin, ^3^mature miRNA sequence used for validation, ^4^estimated probability that a predicted novel miRNA with a score of this or higher is a true positive, ^5^sum of read counts that map to the predicted mature, loop and star miRNAs, and ^6^similar miRNA sequences found in human genome match the novel sequences detected in rhesus monkey.

RNA sequencing enabled the identification of novel miRNAs (Fig. 4). Among the known miRNAs, 13 miRNAs were shared among 4 young muscle tissues (Y1-Y4) (Fig. 4A, *left*), whereas 27 miRNAs were common in 4 old tissues (O1-O4) (Fig. 4A, *middle*). Interestingly, twice as many novel miRNAs were identified in muscle tissues from old monkeys (~ 450) compared to those identified in young tissues (~180) (Fig. 4A, *right*). Complete lists of novel miRNAs in young and old samples are provided (Tables S2 and S3). From this analysis, it appears that several novel miRNAs in monkey muscle tissues have previously been identified in humans. For instance, the precursor coordinates of (chr16:11829814-11829892) and (chr14:109970184-109970240) identified miRNAs that corresponded to the human homologues hsa-miR-744-5p and hsa-miR-34a-5p, respectively (Fig. 4B). In contrast, other novel monkey miRNAs such as those from precursor coordinates (chr18:6923491-6923553) and (chr16:63341749-63341805) did not have any human miRNA homologues (Fig. 4B). These data highlight miRNA sequencing as a method to uncover novel age-associated miRNAs and new human homologues in rhesus monkeys muscle tissues.

### Identification of novel miRNAs in human muscle tissues

**Figure 5:**
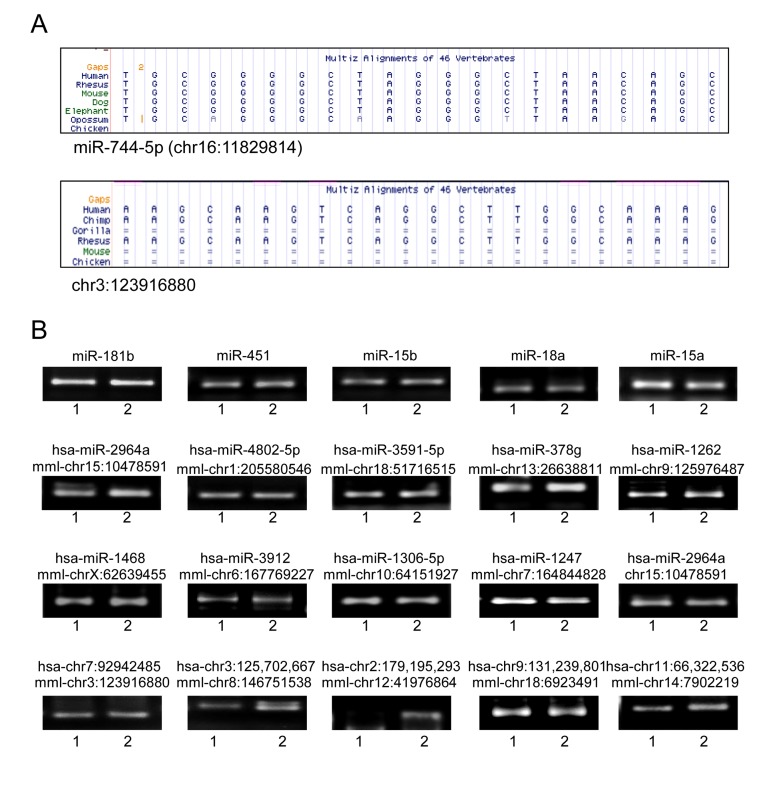
Novel miRNAs in human muscle tissues **(A)** Examples of MULTIZ alignment blocks of chr16:11829814 showing its human homologue miR-744-5p (top) and chr3:123916880 showing similar sequence in human (bottom). **(B)** Representative qPCR products visualized in ethidium bromide-stained agarose gels. These products were obtained using human RNA samples (I,2) from skeletal muscle tissues.

As discussed above, we identified human homologues of novel miRNAs identified in rhesus monkeys using BLAST. Several miRNAs that had not previously been annotated in rhesus monkey appeared to have human homologues. An example is miR-744-5p, which is a novel miRNA in rhesus monkey and is conserved in human and mouse (Fig. 5A, top). In contrast, chr3:123916880 is only conserved in human, rhesus, and chimpanzee (Fig. 5A).

Next, RT-qPCR analysis was performed using total RNA from human adult skeletal muscle tissues in order to confirm that putative novel miRNAs are expressed also in humans. Conserved miRNAs such as miR-181b, miR-451, miR-15b, miR18a, and miR-15 were amplified as positive controls (Fig 5B, *top*) and the human miRNA homologues such as mml-chr1:205580546, mml-chr15:10478591 and mml-chr7:164844828 were identified as well (Fig. 5B, *middle*). Furthermore, novel rhesus monkey miRNAs such as mml-chr3:123916880 and its human homologue hsa-chr7:92942485 were also amplified (Fig. 5B, *bottom*). Thus, a number of novel rhesus monkey miRNAs with known annotation in human genome have been identified in human skeletal muscle along with other miRNAs with no previous annotations.

### Effect of age and CR on novel miRNA expression

**Figure 6:**
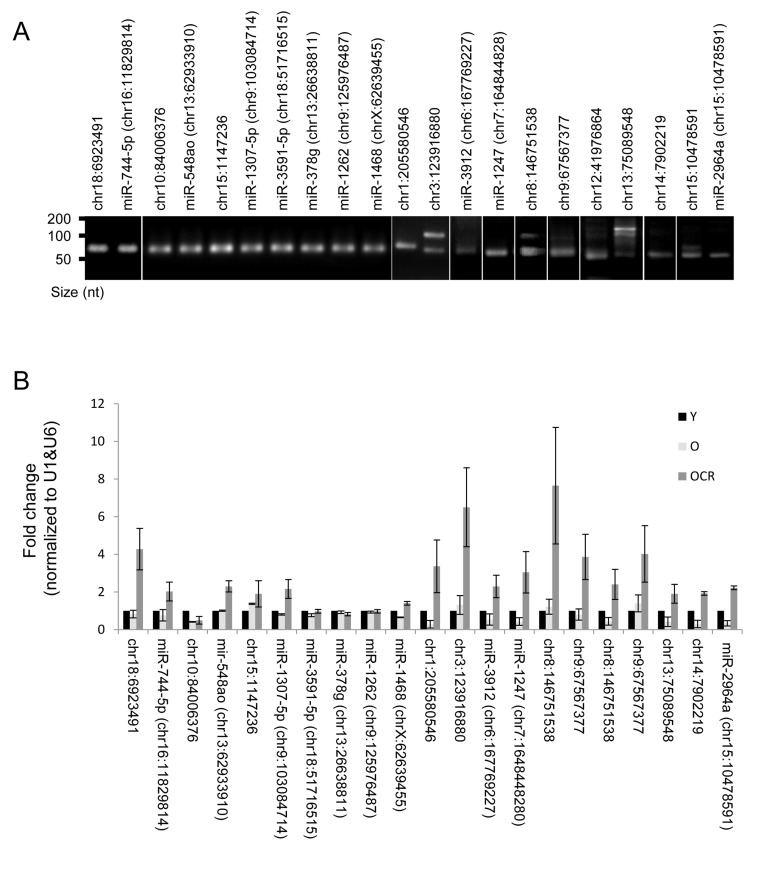
Novel miRNAs and impact of CR **(A)** Representative qPCR products visualized in ethidium bromide-stained agarose gels. These products were obtained using RNA isolated from rhesus monkeys skeletal muscles. **(B)** RT-qPCR analysis of the indicated novel miRNAs and the impact of CR on miRNA expression in OCR samples.

Several novel miRNAs identified in rhesus monkeys were validated by RT-qPCR and the end products were fractionated on agarose gel (Fig. 6A). All tested miRNAs showed single bands at the expected size; however, chr13:75089548, chr8:146751538, and chr3:123916880 showed additional bands in the range of 100 nt, which could represent miRNA precursors. A subset of these miRNAs were altered either by age, CR or both. For instance, chr10:84006376, chr1:205580546, miR-1247, miR-2964a, and chr14:7902219 levels were lower in skeletal muscles of old monkeys. The reduction in expression of some of these miRNAs, e.g., chr1:205580546 and chr14:7902219, was reversed by CR. While the abundance of miRNAs such as chr18:6923491 and chr9:67567377 were not influenced by the age of the monkey, their expression was increased by CR (Fig. 6B). Table S4 depicts a complete list of changes in novel miRNA expression with age. Together, these data indicate that age and CR are factors that can alter expression of several novel miRNAs in skeletal muscle tissues of rhesus monkeys.

## DISCUSSION

Aging encompasses a series of deleterious changes that accumulate in cells and tissues, increasing the risk of diseases and death [18]. Some of these changes are accompanied or driven by age-associated alterations in gene expression [19-21]. Muscle aging is characterized by reduced muscle mass, strength, and ability of regeneration to replace damaged muscle (sarcopenia) [22, 23]. miRNAs are known to play key roles in the regulation of gene expression in a wide range of cellular processes such as proliferation, differentiation, cell survival, death and several other processes [25, 26]. Altered miRNA expression is associated with many biological processes such as cell differentiation, senescence, and division and with pathological conditions including cancer and muscle disorders [17, 27-31].

Profiling of miRNAs provides direct evidence that they are regulated in aged tissues. We previously reported miRNA changes associated with age in peripheral blood mononuclear cells and with replicative senescence of WI-38 human diploid fibroblasts [7, 32]. In the current study, we provide age-associated changes in miRNA expression by sequencing RNAs from young and old skeletal muscle tissues obtained from rhesus monkeys (Table 1 and Table S1). Some of the altered miRNAs are known to be associated with senescence or aging in mouse and human tissues as discussed below.

miR-144 functions in a cluster with miR-451 and both showed the strongest upregulation in old skeletal muscle tissues from rhesus monkeys, as indicated by RNA sequencing and RT-qPCR (Table 1 and Fig. 2). It appears that this cluster is also altered in other aged tissues; for example, the expression of miR-451 is significantly higher in old foreskins compared with young foreskins [33], and miR-451 expression was also elevated in lung tissues of BALB/c old mice [34]. Interestingly, the age-associated increase in miR-144 and miR451 was dampened by CR (Fig. 2), suggesting a protective effect of CR against cellular alterations induced by elevated levels of these miRNAs or perhaps reflected the higher muscle mass in CR monkeys compared to monkeys in normal diet. Interestingly, human subjects who are ‘high responders’ to resistance exercise training, which increases muscle mass, demonstrated lower levels of miR-451 in skeletal muscle than low responders [35]. Together, these miRNAs could be globally altered in several other aged tissues and influenced by exercise training and CR; whether these miRNAs affect other aspects of aging remains to be studied.

The cluster of miR-17-92 includes six members (miR-17, miR-18a, miR-19a, miR-19b, miR-20a, and miR-92a-1); five of them are upregulated in old skeletal muscle tissues (Table 1). It has been reported that miR-18a, miR-19a and miR-19b are downregulated in age-related heart failure [6]. These findings suggest protective effects of these miRNAs in aged muscle tissues and healthy cardiac aging.

While several miRNAs were upregulated in old muscle tissues, a few were downregulated, including miR-181a (Table 1), in agreement with the lower expression of miR-181a in skeletal muscle tissues of old mice [37]. Furthermore, miR-181 has been found to be anti-inflammatory by suppressing the expression of pro-inflammatory cytokines such as TNF-α, IL-6, IL-1β, and IL-8 [38, 39]. It is important to note that an age-associated decrease in miR-181a was also observed in mouse brain but the impact of miR-181a on brain aging is unknown [40]. In aged human subjects, reduced miR-181a was found to be associated with impaired T-cell receptor sensitivity by increasing the activity of dual specificity phosphatase 6 (DUSP6) [41]. Additionally, miR-181a may potentially regulate the type IIA activin receptor, which inhibits proliferation of skeletal muscle-derived progenitors through Smad 2/3 phosphorylation [42]. This observation suggests that downregulation of miR-181a in old muscle tissues may restrict satellite cell proliferation and thus reduce the ability to replace damaged muscle.Thus, we propose that downregulation of miR-181a may be related to increased inflammation in the elderly. However, CR rescued the age-related decline of miR-181a (Fig. 2), which may reflect enhanced muscle mass, strength, and proliferation, as well as reduced inflammation in these animals.

Other miRNAs downregulated with age include miR-489, miR-653, and miR-1323 (Table 1 and Fig. 2c). miR-489 is highly expressed in satellite cells, where it maintains muscle stem-cell quiescence by suppressing the oncogene Dek and thereby preventing the transient proliferative expansion of myogenic progenitors [43]. Short-term CR was recently found to enhance skeletal muscle stem cell function in mice [44], suggesting that the age-related decline in miR-489 could be linked to the reduced pool and functions of satellite cells in old muscle tissues, while CR may restore the levels of miR-489. It is also possible that the higher level of miR-489 in miyoshi myopathy and nemaline myopathy [45] alters muscle cell differentiation and hence tissue repair leading to muscular dystrophy. Future studies are needed to reveal if the age-related decline of miR-489 affects muscle stem cell in the elderly, muscle diseases, and life span.

In addition to differentially expressed miRNAs, sequencing also identified novel miRNAs (Tables S2-S4 and Figs. 4-6). We divided the novel miRNAs in this study into two groups. The first group includes novel miRNAs that have annotated human homologues, whereas the second group consisted of novel miRNAs that have no match with known miRNAs. We also present evidence that the un-annotated novel miRNAs could be expressed in human skeletal muscle tissues indicating that similar miRNAs could be identified in humans (Fig. 5). Validation by RT-qPCR indicated that age and CR can alter their expression (Fig. 6). The regulatory mechanisms responsible for the altered microRNA levels in old and young muscles are not known at this time, but they likely include arrays of transcriptional and post-transcriptional regulatory factors, as reported for other age and senescence-associated gene expression patterns [46-51].

In summary, we have identified age-associated alterations in miRNA expression of skeletal muscle tissues from rhesus monkeys. We also identified collections of novel miRNAs both in young and old muscle tissues and showed that age-related changes of known and novel miRNAs are influenced and in some cases reversed by CR. Future studies will help to recognize the roles of these changes, including novel miRNAs, in life span and age-related muscle diseases.

## MATERIALS AND METHODS

### RNA isolation, miRNA sequencing and IPA analysis

Skeletal muscle tissues obtained from 9 young (Y), 7 old (O) and 9 caloric restricted old (OCR) rhesus monkeys were homogenized in Trizol (Invitrogen, NY, USA) and used to isolate total RNA according to manufacturer's protocol. Total RNAs samples from human adult normal skeletal muscle tissues were obtained from Biochain (Newark, CA, USA). These RNA samples included RNA sample from one donor (R1234171-50) and a mixture of RNA samples from five donors (R1234171-P).

Total RNA from rhesus monkeys were used for miRNA sequencing or validation by RT-qPCR (details below). The TruSeq Small RNA kit (Illumina, San Diego, CA, USA) was used to isolate miRNAs from young and old total RNA according to manufacturer's protocol. Briefly, adapters specific to the 5'-phosphate and 3'-hydroxyl group were ligated to total RNA. The ligation product was then reverse transcribed and amplified using indexed primers. The resulting cDNA constructs were size-selected using *Polyacrylamide gel* electrophoresis (PAGE) and concentrated by ethanol precipitation. The miRNA library was validated and quantified on an Agilent Technology 2100 Bioanalyzer. Indexed libraries were pooled prior to clustering on an Illumina TruSeq flowcell and then sequenced. The results of differentially expressed miRNAs were subjected to Ingenuity Pathway Analysis (IPA) to determine possible participation of these changes in biological processes.

### cDNA synthesis, RT-qPCR, DNA gel electrophoresis and imaging

QuantiMir RT Kit (System Biosciences, CA, USA) was used to validate miRNA sequencing data. Mature known and novel miRNAs as well as U1 and U6 small nuclear RNAs (snRNAs) were detected from skeletal muscle total RNA. Briefly, all cellular RNA was polyadenylated using poly(A) polymerase at 37°C for 10 min, thus enabling the subsequent annealing of a oligo-dT adaptor for 5 min at 60°C. The miRNA levels of miRNAs were assessed by RT-qPCR using the SYBR green PCR master mix (Kapa Biosystems, MA, USA), a universal reverse primer, and miRNA-specific forward primers obtained from Integrated DNA Technologies CA, USA (see below). RT-qPCR analysis was performed on Applied Biosystems (NY, USA) model 7300 and 7900 instruments and the averaged U1 and U6 snRNA levels were used for normalization. Specific miRNA forward primers were as follows: TACAGTATAGATGATGTACT for miR-144; TAGCAGCACATAATGGTTTGTG for miR-15a; TAGCAGCACATCATGGTTTACA for miR-15b; TAAAGTGCTGACAGTGCAGAT for miR-106b; AAGCCCTTACCCCAAAAAGTAT for miR-129-3p; CTTTTTGCGGTCTGGGCTTGC for miR-129-5p; TAAGGTGCATCTAGTGCAGATA for miR-18a; AATACTGCCGGGTAATGATGGA for miR-200c; TGGCAGTGTCTTAGCTGGTTGT for miR-34a; TGTCAGTTTGTCAAATACCCC for miR-223; GAATGTTGCTCGGTGAACCCCT for miR-409-3p; AAACAAACATGGTGCACTTCTT for miR-495; AAACCGTTACCATTACTGAGTT for miR-451; TCAAAACTGAGGGGCATTTTC for miR-1323; AACATTCATTGCTGTCGGTGGGTT for miR-181b; GTGTTGAAACAATCTCTACTG for miR-653; GTGACATCACATATACGGCAGC for miR-489; AGGAGTGACAGAGTTAGACTC for chr18:6923491; TGCGGGGCTAGGGCTAACAGC for miR-744-5p; ACGATAACACCCAAGTCCGAA for chr10:84006376; AAAAACCGTAGTTACTTTTGC for miR-548ao; TGCTGTCCCTCCAGAGCCGTGGC for chr15: 1147236; TCGACCGGACCTCGACCGGC for miR-1307-5p; TTTAGTGTGATAATGGCGTT for miR-3591-5p; ACTGGGCTTGGAGTCAGAAGAC for miR-378g; TTGGGTGACATTTGTAGATG for miR-1262; CTCCGTTTGCCTGTTTTGCTGA for miR-1468; TCTGATCGTTCCCCTCCATACA for chr1:205580546; AAGCAAGTCAGGCTTGGCAAAG for chr3:123916880; UAACGCAUAAUAUGGAUAU GU for miR-3912; AAAAGGAATTGCGGTTTTTGA CA for miR-548an;ACCCGTCCCGTTCGTCCCCGA for miR-1247; CCTGTTTGTCTGAAATTCAAAT for chr8:146751538; TCCCCCAGTCTGGCCACAGAGC for chr9:67567377; TCTGTGCAGAACTCATAGAAG for chr12:41976864; TCCTGGGCTTTGGCAGACAGC for chr13:75089548; CCCCCGTCTCTCTCTGTTCAG for chr14:7902219; and AGATGTCCAGCCACAATT CTCG for chr15:10478591. U1 and U6 forward primer sequences were CACCACGTTTATACGCCGGTG and CGACTGCATAATTTGTGGTAGTGG respectively. RT-qPCR products were size-separated using 4% ethidium bromide agarose gels and visualized on an ultraviolet transilluminator EL LOGIC 1500 imaging system.

### Animals

Rhesus monkeys (*Macaca mulatta*) were housed continuously at the NIH Animal Center, Poolesville, MD. The animal center is fully accredited by the American Association for Accreditation of Laboratory Animal Care, and all procedures were approved by the Animal Care and Use Committee of the NIA Intramural Program. Monkeys were housed individually in standard nonhuman primate caging on a 12h light/12h dark cycle, room temperature 25.5 ± 0.5 °C, humidity at 60 ± 20%. Monkeys received 2 meals per day at estimated *ad libitum* levels throughout the study and water was always available. Monkeys were monitored minimally 3 times daily by trained animal care staff. Young and old monkeys were obtained from an ongoing NIA longitudinal study on dietary manipulation and aging that began in 1987 [52, 53]. We used tissues obtained from 9 young (Y, ~6 years of age), 7 old (O, ~26.8 years of age), and 9 old caloric restricted (OCR, ~28.8 years of age), all males. While Y and O monkeys were maintained on normal diet (TestDiet® #5038 Purina Mills, Richmond, IN), OCR animals were maintained on caloric restricted diet (TestDiet® #5L1F, Purina Mills, Richmond, IN) most of their lives (20.8-22.6 years).

## SUPPLEMENTARY TABLES









## Abbreviations

ALS: Amytrophic Lateral Sclerosis
CR: caloric restriction
DMD: Duchenne muscular dystrophy
IL: interleukin
MD: Muscular Dystrophy
RISC: RNA-induced silencing complex
TNFα: tumor necrosis factor alpha
